# Adrenal ectopy and lipoma of an inguinal hernia sac: A case report & literature review

**DOI:** 10.1016/j.ijscr.2020.12.075

**Published:** 2020-12-28

**Authors:** Jenna R. Adalbert, Rafael E. Pajaro

**Affiliations:** a Sidney Kimmel Medical College at Thomas Jefferson University Hospital, 1025 Walnut Street #100, Philadelphia, PA, 19107, USA; b Morristown Medical Center of Atlantic Health System, 100 Madison Avenue, Morristown, NJ, 07960, USA

**Keywords:** Ectopic adrenal, Inguinal hernia repair, Hernia sac, Lipoma, Adrenocortical tissue

## Abstract

**Introduction & importance:**

Ectopic adrenocortical tissue is the presence of accessory adrenal cortex tissue located outside of the adrenal glands. It is a rare, incidental finding during inguinal hernia repair. This case report aims to discuss the anatomy and important patient implications related to this finding.

**Case presentation:**

A 61-year-old male presented with a long-standing right direct inguinal hernia increasing in size and pain frequency. During open right anterior inguinal hernia repair, a lipoma was identified inside the hernia sac and removed. Further histopathological examination of the specimen revealed the presence of adrenocortical tissue inside the lipoma. No further interventions were performed. The postoperative and 2-year follow-up course were uneventful.

**Clinical discussion:**

Adrenocortical tissue can parallel the descent of the gonads during embryogenesis, arresting at any point along this path, including the inguinal region. Ectopic adrenocortical tissue is commonly found during inguinoscrotal procedures in infants, suggesting early involution. Its incidence in hernia sacs should be recognized to prevent misdiagnosis as neuroendocrine tumors or melanomas. If adrenocortical tissue is identified during hernia reduction, further surgical exploration is not recommended. In terms of prognosis, endocrine imbalances following surgical removal cannot be ruled out, promoting careful patient monitoring. The lipoma containing the adrenal tissue in our case is a common finding during hernia repair, identified in 22% of patients at operation.

**Conclusion:**

Ectopic adrenocortical tissue is a rare, incidental finding during inguinal hernia sac analysis in adults. This finding should be well-documented following removal and further studies are required to evaluate long-term outcomes.

## Highlights

• Ectopic adrenocortical tissue is accessory adrenal cortex tissue outside of the adrenal glands.
• Ectopic adrenocortical tissue is a rare finding during inguinal hernia repair in adults.
• Adrenocortical tissue in inguinal hernia sacs can mimic metastatic deposits.
• Surgical excision of adrenocortical tissue may have endocrine implications.
• We have described a case of adrenocortical tissue identified in a lipoma of an inguinal hernia sac.

## 1 Introduction

Ectopic adrenocortical tissue is the presence of accessory adrenal cortex tissue located outside the suprarenal location of the adrenal glands [1]. It is a rare, incidental finding during inguinal hernia repair in the adult male population [2]. Given the proximity of adrenocortical tissue to the gonads during embryogenesis, it may parallel gonadal descent and arrest at any point along the migration pathway, including the inguinal region [3]. While no evidence-based guidelines exist for management following surgical excision during hernia repair, removal of endocrine tissue may require special considerations. Herein we report the case of ectopic adrenocortical tissue identified within a lipoma of an inguinal hernia sac, followed by a discussion of patient implications. This work has been reported in compliance with the SCARE 2020 criteria [4].

## 2 Case presentation

A 61-year-old male presented for surgical consultation of a long-standing right direct inguinal hernia that had increased in size and intensified in pain frequency over the past two months. He denied any symptoms of bowel obstruction or endocrine derangements. His past medical history was inclusive of well-controlled hypertension with Valsartan and recently diagnosed prostate cancer being observed without therapy. Family history was non-significant. On physical examination, the hernia was readily reducible.

An open right anterior inguinal hernia repair with mesh was performed under local anesthesia with sedation by a senior, board-certified general surgeon at a major academic medical center. With the patient in supine position, a transverse incision was made over the inguinal canal and superficial veins were ligated with 3−0 silk. Scarpa’s fascia and the external oblique over the course of its fibers to the superficial ring were opened. The spermatic cord was mobilized with a Penrose drain and dissected out. During dissection, a large lipoma of the cord was identified, high ligated with 3-0 silk, and removed to optimize repair. A direct hernia was subsequently reduced off the bottom of the cord. Repair was performed with a Prolene mesh patch sewn in place with 2-0 Prolene to the inguinal ligament and anterior rectus sheath, as well as 2-0 PDS to the pubic tubercle region. The previously dissected cord and ilioinguinal nerve were placed through a slit in the mesh and it was re-closed with a running 2-0 Prolene to recreate the deep ring. Tails of the mesh were tucked laterally and medially over the transversus arch, and inferiorly over the pubic tubercle to prevent recurrence. The external oblique was closed with 3-0 Vicryl, Scarpa’s with 3-0 plain, and the skin with 4-0 Monocryl subcuticular suture and Dermabond.

**Fig. 1:**
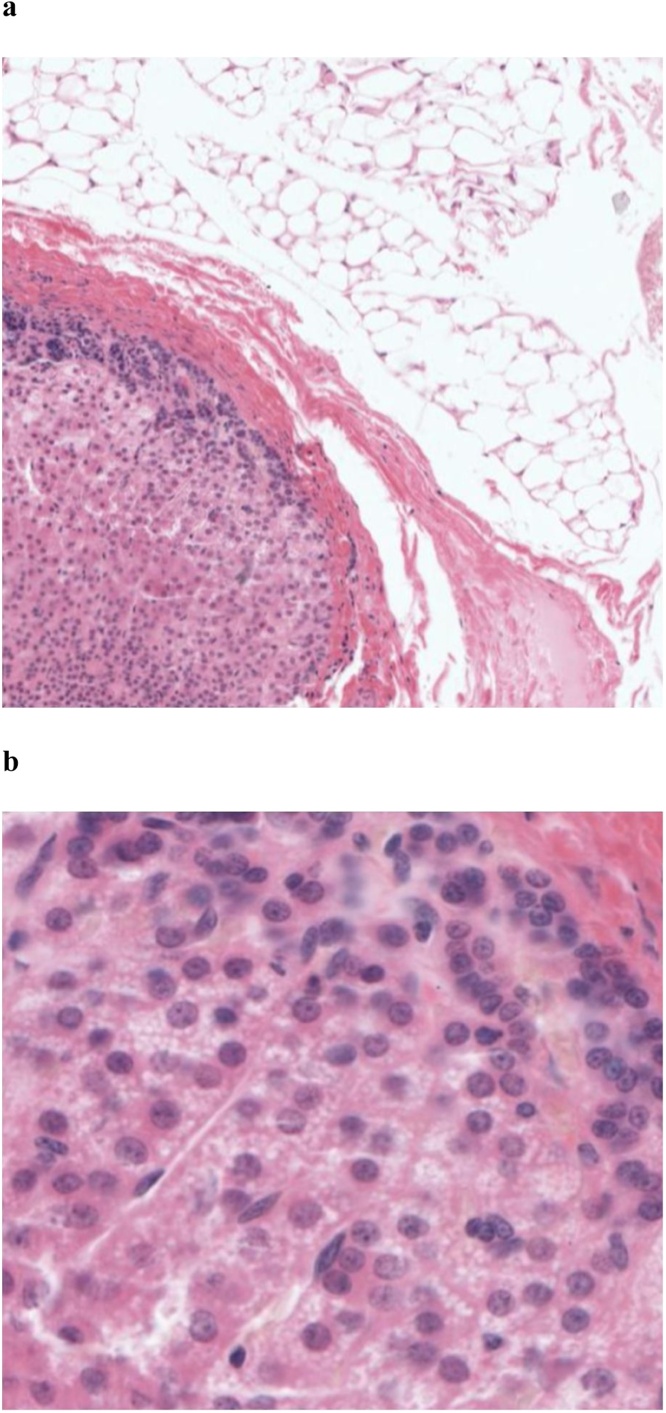
Encapsulated adrenocortical tissue surrounded by benign fibroadipose tissue in a hernia sac, (a) low power view, (b) high power view.

Gross examination of the lipoma specimen revealed an irregular fragment of yellow-tan, lobulated fibroadipose tissue covered by a semi-translucent fibrous capsule and measuring 4.7 × 2.0 cm. Upon sectioning, a central tan-orange, rubbery nodule measuring 0.5 cm in greatest dimension was discovered amid the fatty exterior. Further histopathological examination was consistent with adrenocortical tissue displaying the customary zonation pattern of cells surrounded by a well-defined fibrous capsule and layers of fibroadipose tissue (Fig. 1). No adrenal medullary tissue or dysplastic changes were identified upon subsequent submission of the entirety of the specimen. The final case diagnosis was reported as *a hernial sac containing a benign “lipoma of the cord” with accessory adrenal gland tissue*. Our patient received no further interventions and has had no significant changes in health throughout the 2-year follow-up period.

## 3 Discussion

During inguinal hernia sac analysis, ectopic adrenal tissue is a rare, incidental finding in adults [2]. To the extent of our knowledge, there have only been ten previously documented cases in English literature with all occurring exclusively in male patients [5]. However, it is not difficult to hypothesize the mechanisms of this ectopia when considering the migration of tissue during embryogenesis. Adrenocortical tissue is derived from the mesoderm medial to the developing gonads [1] and can parallel the descent of gonadal tissue with the potential to arrest at any point along this path. Consequently, it has been identified around the celiac axis (32%), broad ligament (23%), adnexa of the testes (7.5%), and spermatic cords (1–9.3%) [3]. In contrast to the adult population, the incidental finding of ectopic adrenocortical tissue during inguinoscrotal procedures in the infantile pediatric population has been extensively documented, suggesting that this tissue typically involutes during the first year of life [5]. Considering embryologic origins, the absence of literature describing ectopic adrenal *medullary* tissue (in contrast to ectopic adrenal *cortical* tissue) in these locations is predictable since it is derived from the neuroectoderm with less proximity to the gonads [1,2].

Of note, ectopic adrenocortical tissue is not the only anatomically aberrant specimen procured during hernial sac analysis. Additional findings have reported various metastases, endometriosis, undescended testicles, bladder tissue, appendices, glandular inclusions from Wolffian or Mullerian remnants, and subsequent mesothelial hyperplasia from trauma or inflammation [5]. When performing histopathological review of hernia sac specimens, it is important to recognize the rare occurrence of adrenocortical ectopy in order to prevent misdiagnoses as metastatic deposits and spare patients unnecessary testing. Morphologic and immunohistochemical characteristics of adrenocortical tissue may mimic well-differentiated neuroendocrine carcinomas (i.e. carcinoid) or metastatic melanomas by displaying reactivity to markers such as synaptophysin and CD-56 or MART-1 [5].

As with any originally benign tissue, the growth and evolution of adrenocortical ectopy may have negative sequelae for patients. Following therapeutic bilateral adrenalectomy for pathologic ACTH production, hyperplasia at a site of ectopic adrenal tissue could potentially precipitate disease recurrence. Neoplastic developments of ectopic adrenal nodules have also been reported in the literature and may be important to consider in the differential during investigation of endocrine imbalances [5]. Following surgical excision of the ectopic adrenocortical tissue, further considerations regarding prognosis should include the possibility of adrenal insufficiency. It is fair to hypothesize that homeostatic endocrine mechanisms may be disrupted if the tissue is either functional in the absence of normal adrenal glands or hyperfunctional with suppression of the hypothalamic-pituitary-adrenal axis [3].

In reality, the true incidence of adrenal ectopy in the inguinal region is likely underestimated as it is related to incidental findings during surgery. While excision of the ectopic adrenocortical tissue is usually easy to achieve, routine intraoperative searches of the spermatic cord are not recommended due to the risk of disrupting the native anatomy [3]. Further surgical exploration beyond the requirements of adequate hernia reduction may result in irreversible injury to the spermatic vessels or vas deferens [1,3].

Curiously, our ectopic adrenocortical tissue was enveloped in a significantly sized spermatic cord lipoma. Lipomas are a common finding during hernia repair, reportedly identified in 22% of patients at the time of operation [6]. These benign fatty masses should be recognized as a clinically significant entity due to their potential to produce hernia-like symptoms in the absence of a true hernia. They may also result in unsatisfactory postoperative results if they are not thoroughly removed during reduction [6]. While ultrasonographic evaluation is typically effective at differentiating testicular carcinoma from lipoma, unclear sonographic findings and a high symptomatic suspicion of lipoma (i.e. slowly growing, non-reducible, fixed, relatively hard, nontender mass) should prompt further preoperative modalities of investigation such as CT or MRI for diagnostic confirmation [7].

## 4 Conclusion

Ectopic adrenocortical tissue is a rare, incidental finding during inguinal hernia sac analysis in adults. In the present case, surgical excision of ectopic adrenocortical tissue identified inside of an inguinal hernia sac during repair has not been followed by any significant changes in patient health over a 2-year period. However, this finding should be well-documented for patient monitoring and further studies are required to evaluate long-term outcomes.

## Declaration of Competing Interest

There are no conflicts of interest to report.

## Funding

None.

## Ethical approval

On the basis of this being a single case report and not a case series, the Institutional Review board of Thomas Jefferson University does not mandate that ethical approval is required. Therefore, this case report is considered exempt.

## Consent

Written informed consent was obtained from the patient for publication of this case report and accompanying images. A copy of the written consent is available for review by the Editor-in-Chief of this journal on request.

## Author contribution

Case description, case discussion, and literature review: Jenna R. Adalbert, BS.

Advised and final proofreading of the report: Rafael E. Pajaro, MD, MS.

## Registration of research studies

Not applicable.

## Guarantor

Rafael Pajaro, MD, MS.

## Provenance and peer review

Not commissioned, externally peer-reviewed.
